# Microflow injection analysis based on modular 3D platforms and colorimetric detection for Fe(III) monitoring in a wide concentration range

**DOI:** 10.1007/s00604-023-06029-x

**Published:** 2023-12-02

**Authors:** David Ricart, Antonio David Dorado, Conxita Lao-Luque, Mireia Baeza

**Affiliations:** 1 Universitat Politècnica de Catalunya, Avinguda de les Bases de Manresa 61-73, 08240 Manresa, Spain; 2 GENOCOV Research Group, Department of Chemistry, Faculty of Science, Edifici C-Nord, Universitat Autònoma de Barcelona, Carrer dels Til·Lers, 08193 Bellaterra, Spain

**Keywords:** Fe(III), 3D-printed microfluidic platform, FIA, Bioleaching, e-waste, Circular economy

## Abstract

A modular microflow injection analysis (microFIA) system for the determination of Fe(III) in a bioleaching reactor has been designed, developed and validated. The different modules of the analyzer (mixer, diluter, disperser and detector) were 3D-printed. Fe(III) quantification is due by measuring the color intensity of the chelate formed between Fe(III) and salicylic acid at 525 nm. The device has been designed to dilute, disperse and detect high Fe(III) concentrations in the form of an inexpensive multi-step photometric flow cell that uses an light-emitting diode (LED) as a light source and an light-dependent resistor (LDR) as a light intensity detector. This microFIA system has been shown to be suitable for automatic and continuous determination of Fe(III) in the operation of a bioreactor for the oxidation of Fe(II). The device has a good repeatability (less than 5% of coefficient of variation in the whole range of concentrations) and accuracy of around 100%. The analyzer features an exceptional wide linear range, between 25 and 6000 mg·L^−1^. The device was successfully applied to the determination of Fe(III) in real samples. The obtained results proved that the method is applicable for accurate, precise, rapid, and low-cost colorimetric analysis and didn’t show significant differences with a conventional UV–Vis method.

**Graphical Abstract:**

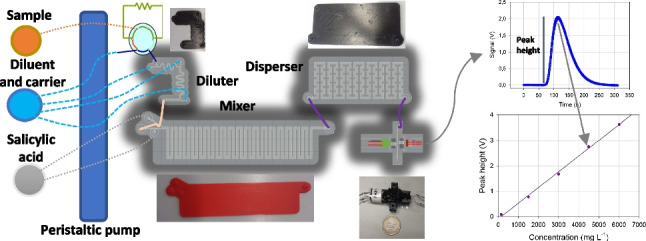

**Supplementary Information:**

The online version contains supplementary material available at 10.1007/s00604-023-06029-x.

## Introduction

In recent decades, the combination of rapid technological advances and obsolescence has increased e-waste by 3–5% per year according to the World Health Organization (WHO) [1]. Consequently, it has become increasingly urgent to find methods for recycling e-waste not only for environmental reasons but also because e-waste typically contains valuable metals such as copper, silver, cobalt, lithium, gold, nickel, among other and so, their recovery for new uses is highly profitable.

However, e-waste recycling is difficult due to its complex structure and heterogeneous blend of organic materials, ceramics, and metals. Currently, pyrometallurgical and hydrometallurgical methods are the primary processes utilized for the recovery of these metals from e-waste. Nonetheless, these methods entail substantial economic costs and have a significant environmental impact due to their use of high temperatures and harsh chemicals. Recently, other recycling technologies that are more environmentally sustainable have been under investigation. Among these is the bioleaching process, which is based on the activities of certain microorganisms that generate a leaching agent responsible for metal extraction. Bioleaching is currently employed in the mining industry for low-grade ores [2], such as in bio-heap processes for commercial applications [3, 4], but it may also be effective in the industrial-scale recovery of metals from e-waste [5, 6].

*Acidithiobacillus ferrooxidans* has been employed for the bioleaching of copper from electronic waste via the oxidation capacity of Fe(III) at acidic conditions. The bacteria possess the ability to re-oxidize Fe(II) into Fe(III) following the oxidation and solubilization of the copper content in the matrix, thus enabling a cyclic process to be established [7]. The copper bioleaching process is typically executed in two stages. In the initial stage, the oxidation process transpires within a bioreactor where microorganisms oxidize Fe(II) to Fe(III). Following this, the Fe(III) solution, without the presence of bacteria, is introduced into the reactor containing the e-waste where Cu(II) extraction occurs. Monitoring the concentrations of Fe(II) or Fe(III) is crucial in both stages for controlling and optimizing the process. Thereby, a fast and accurate technique for measuring these ions would prove beneficial in researching, managing, and overseeing these processes.

Various instrumental techniques have been proposed to analyze Fe(III) in water samples, such as atomic absorption spectrometry [8], inductively coupled plasma-mass spectrometry [9], inductively coupled plasma-optical emission spectrometry [10], fluorescence spectroscopy [11, 12], as well as cathodic or anodic stripping voltammetry [13] and chromatography [14]. However, colorimetric analysis of iron ions is considered a particularly significant, attractive, and straightforward method. A myriad of chelating reagents of Fe(III) have been proposed in the literature. One of the most commonly employed colorimetric reagent is 1,10-phenanthroline [15, 16]. To determine ferric iron, it is necessary to reduce it to Fe(II) using reducing agents like hydroxylamine hydrochloride. The use of a phenanthroline derivative, bathophenanthroline, has also been suggested for the determination of ferrous iron [17]. A new approach for concurrently measuring ferrous and ferric iron concentration in aqueous samples was presented by Karamanev [18]. This method involves determining both ferric and total iron simultaneously at distinct light wavelengths, thereby substantially reducing analytical procedure errors. The technique relies on the colorimetric assessment of a red-coloured ferric-sulfosalicylate complex formed under acidic conditions that absorbs at 500 nm. Following the addition of ammonia, which raises the pH, 5-sulfosalicylic acid (SSA) forms a yellow complex with all iron ions in the solution it has highest light absorbance at 425 nm. More recently, novel chelating reagents have been used to analyze Fe(III) by colorimetry such us deferiprone [19], benzimidazole [20], 4-mercaptophenol and mercaptoacetic acid [21], glycine [22], 8-hydroxyquinoline [23], gold nanoparticles [24, 25], 2,5,7-triarylimidazopyridine [26] and ferrozine [27].

However, a major drawback of many colorimetric methods is that they are highly manual and non-automated, requiring trained personnel, controlled laboratory environments, and in many cases, prior sample treatment, which affect analysis time and quality. Furthermore, each measurement made using colorimetric methods requires a high consumption of sample, reagents, and time, which is a significant limitation in monitoring bacterial activity inside the bioreactor over time and limits the number of measurements. These constraints render these methods unsuitable for on-site monitoring.

Most of these problems and limitations can be overcome by flow injection analysis (FIA). The FIA technique has the capability to monitor various parameters, including Fe ions, in different matrices [14, 28–31]. FIA methods provide real-time and in situ analysis, allowing for automation and high sampling throughput. However, there is currently a lack of published research regarding the monitoring of Fe(III) concentrations in a bioreactor using FIA. Additionally, the linear range response of these methods is quite narrow, so large dilutions must be made when analyzing high concentration samples, which is often a source of error for Fe(III) concentration. The linear range found in similar works is 0.05–4.0 mg·L^−1^ [32], 1–40 mg·L^−1^ [29], 0.2–5 mg·L^−1^ [33], 0.021–2.4 mg·L^−1^ [34], 40–350 mg·L^−1^ [35] and 110–560 mg·L^−1^ [36].

Accordingly, the objective of this study is to design, develop, and validate a reliable and adaptable microFIA system, fully automated and integrated, utilizing 3D printing technology for on-site monitoring a wide range of Fe(III) concentrations in a bioreactor. The detection of Fe(III) is achieved through the development of a coloured complex between the iron ion and salicylic acid which is specific for Fe(III) at acidic pH [37] that absorbs at 525 nm. The device will be designed using a CAD program, featuring narrow microchannels that require minimal volumes of both samples and reagents. These features will enable cost savings and frequent, on-site, automatic and reliable determination of Fe(III) in a broad range of concentrations.

## Materials and methods

### Chemicals and reagents

In this study, we utilized a polylactic acid (PLA) filament of 1.75 mm produced by LEON 3D (Valverde de la Virgen, Spain) in three different colours: black, white and transparent. We also used FeNH_4_(SO_4_)_2_·12H_2_O (99%), salicylic acid (99.5%), and ethanol (96%), all of which were obtained from Scharlab (Barcelona, Spain). H_2_SO_4_ (95–98%) was purchased in Panreac (Castellar del Vallès, Spain). For the selectivity study, a solution of 1000 mg·L^−1^ of Cu(II) was obtained from CuSO_4_·5H_2_O (Labkem, Barcelona, Spain), a 1000 mg·L^−1^ of Al(III) from Al_2_(SO_4_)_3_·18H_2_O (Scharlab, Barcelona, Spain) and 1000 mg·L^−1^ of Fe(II) from FeSO_4_·7H_2_O (Chem-lab, Barcelona, Spain). Additionally, we utilized a red phenol dye from Scharlab (Barcelona, Spain). All chemicals utilized in this study were of analytical reagent grade.

To prepare Fe(III) solutions, an aqueous acidic solution at pH = 1.8–2.0 was prepared by dissolving H_2_SO_4_ in deionized water. For FIA experiments, a stock solution of Fe(III) with a concentration of 6000 mg·L^−1^ was prepared. For the UV–Vis assays, a Fe(III) stock solution with a concentration of 375 mg·L^−1^ was prepared by dissolving FeNH_4_(SO_4_)_2_·12H_2_O in the acidic solution. It was critical to maintain a pH value of 2.0 or below throughout the experiment to prevent the precipitation of Fe(OH)_3_ inside the channels.

The chelating reactant solution was prepared by dissolving salicylic acid in ethanol at a concentration of 5% (w/v) for its use in UV–Vis experiments and a 0.180% (w/v) solution was prepared for FIA experiments using the aqueous acidic solution. The acidic solution was obtained by adding H_2_SO_4_ (1:1) to any volume of deionized water, stirring continuously, until a pH of 1.8 for batch and pH of 2.0 for FIA was reached. Salicylic acid is known to form a coloured purple complex with Fe(III), which exhibits an absorption peak at 525 nm.

A 1% solution of a red phenol dye in water was prepared to check the tightness of the modules and the correct functioning of the detector. All solutions were prepared with deionized water for Milli-Q system (Millipore, Billerica, MA, USA).

### Microfluidic 3D modules design, printing conditions and fabrication

The FIA-designed system consisted of four 3D-printed microfluidic modules: diluter, mixer, disperser, and detector. The CAD model design of the four modules was developed using AutoCAD (Autodesk). A Prusa i3MK3S + printer was used for the manufacturing of all the pieces (Prusa Research Prague, Czech Republic).

The PLA was chosen to print modular blocks because it is not expensive, eco-friendly, and has mechanical characteristics suitable for the purpose of this work. Black, white and transparent PLA were tested to find the best colour for pieces. The models of the different pieces were exported as STL files to CURA software where the printing settings were defined. The files were then exported as gcode files to SD targets and the codes were read by a 3D-printer.

The printing conditions for the PRUSA printer involved a layer height of 0.1 mm, with the first layer set to a height of 0.2 mm. Line width 0.2 mm, top/bottom thickness 1 mm, wall line count 5, main speed 60 mm/s, 10 bottom and top layers, initial printing temperature was 220 °C and thereafter 215 °C, plate temperature was 60 °C and a retraction distance of 1.6 mm. During the CAD design process, a distance of 4–5 mm was deemed sufficient between the channel and the external wall.

In order to test for leaks, several pieces were 3D-printed using transparent PLA. The red phenol dye was then passed through the channels in these pieces to detect any potential leaks.

### Experimental setup

**Fig. 1:**
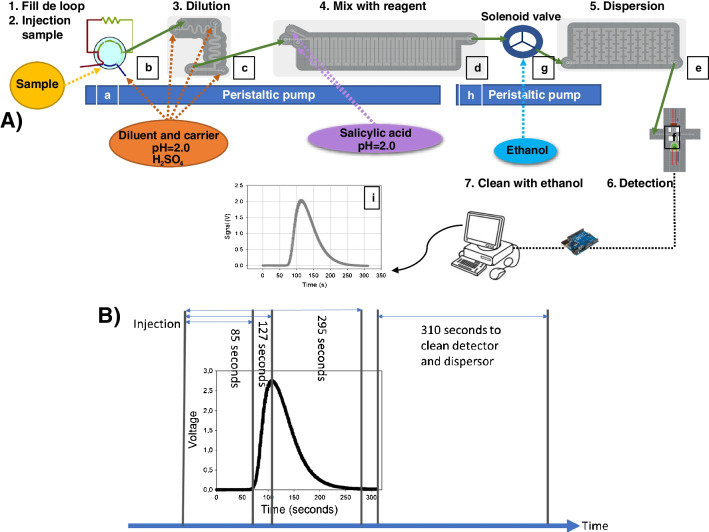
(**A**) Scheme of the microFIA system to determine Fe(III). (a) Peristaltic pump for solutions, (b) six-way valve, (c) diluter, (d) mixer, (e) disperser, (f) detector, (g) 3-way solenoid valve, (h) peristaltic pump for cleaning, (i) analytical signal, the peak height is proportional to concentration. (**B**) Analytical signal and sequence of time diagram to determine Fe(III): injection time, start-up time, time at the maximum signal, and recovery time of the baseline. The total analysis time is 10.1 min

The scheme of the complete microflow injection analysis (microFIA) device is shown in Fig. 1A. The main parts are: (i) the injection system, (ii) the four 3D-printed modules assembled (diluter, mixer, disperser and detector) and (iii) the acquisition data system.

Fluids flow (sample, carrier, reactant) was managed by two active components, peristaltic pumps and valves. A GILSON Minipuls 3 R8, with an 8-channel head, (Middleton, WI, USA) peristaltic pump equipped with PVC tubing with 1.52 mm internal diameter Tygon®tubing (Ismatec, Swiss) was used to pump the sample, reagent and H_2_SO_4_ solution as diluent and carrier. For the sample injection, a six-way injection valve manufactured by Hamilton Injector MVP (Cary, NC, USA) was utilized.

The experimental operational parameters were fixed as follow. Flow rate of solutions was between 0.33 and 0.41 mL/min. The carrier flow rate was 0.33 mL/min, the diluent flow rate was 0.39 mL/min, the reagent flow rate was 0.36 mL/min, and the ethanol flow rate was 1.36 mL/min. The injection volume was 25 µL, and the volume of the diluter, mixer and disperser was 95.1 mm^3^, 2034 mm^3^ and 1524 mm^3^, respectively.

The detection system was manufactured by assembling a 525 nm light-emitting diode (LED) green (OSGGD25112A, Transfer Multisort Elektronik, Polonia) and a light-dependent resistor (LDR) (PGM5537, Transfer Multisort Elektronik, Polonia), to serve as a detector, connected by Arduino UNO with PC via USB. The signal acquisition software by LabVIEW was custom made to this detection system.

To clean the detector, a 3-way solenoid valve 161T031 from NResearch (West Caldwell, NJ, USA) and a Dinko D-21 V peristaltic pump (Dinko instruments, Barcelona, Spain) was used. The solenoid valve was connected to an interface Cool Drive Valve Drivers for 161 series/12 vdc., 161D5X12 of NResearch (West Caldwell, NJ, USA) to carry ethanol inside for detector cleaning between subsequent measurements.

The four 3D printing modules (diluter, mixer, disperser, and detector) of the microFIA system were joined with PVC tubing and polytetrafluoroethylene (PTFE) tubes (Tecator, Hohganes, Sweden) of variable length and 0.8 mm internal diameter.

The analytical signal obtained from the system is shown in Fig. 1Ai. The height of the peak is proportional to Fe(III) concentration. The total analysis time is 605 s (10.1 min). In detail, the partial times are 0 s injection time, 85 s start-up time, 127 s peak maximum height, 295 s recovery time of 95% of the baseline value and 310 s clean step (Fig. 1B).

The inlet/outlet 3D-printed modules were designed as described in a previous study [34]. Different sizes of inlet/outlet systems were tested in order to ensure complete tightness. Finally, the external diameter was 2.72 mm, the internal diameter was 1.88 mm and the diameter on the bottom was 1.02 mm but the last one will vary according to the module. Due to these dimensions, the connecting tubes had to be expanded to be able to fit them into the inlet and outlet of the platform to ensure no leaks. The isolated structure is shown in Fig. S1.

### 3D-optical flow-cell detector

**Fig. 2:**
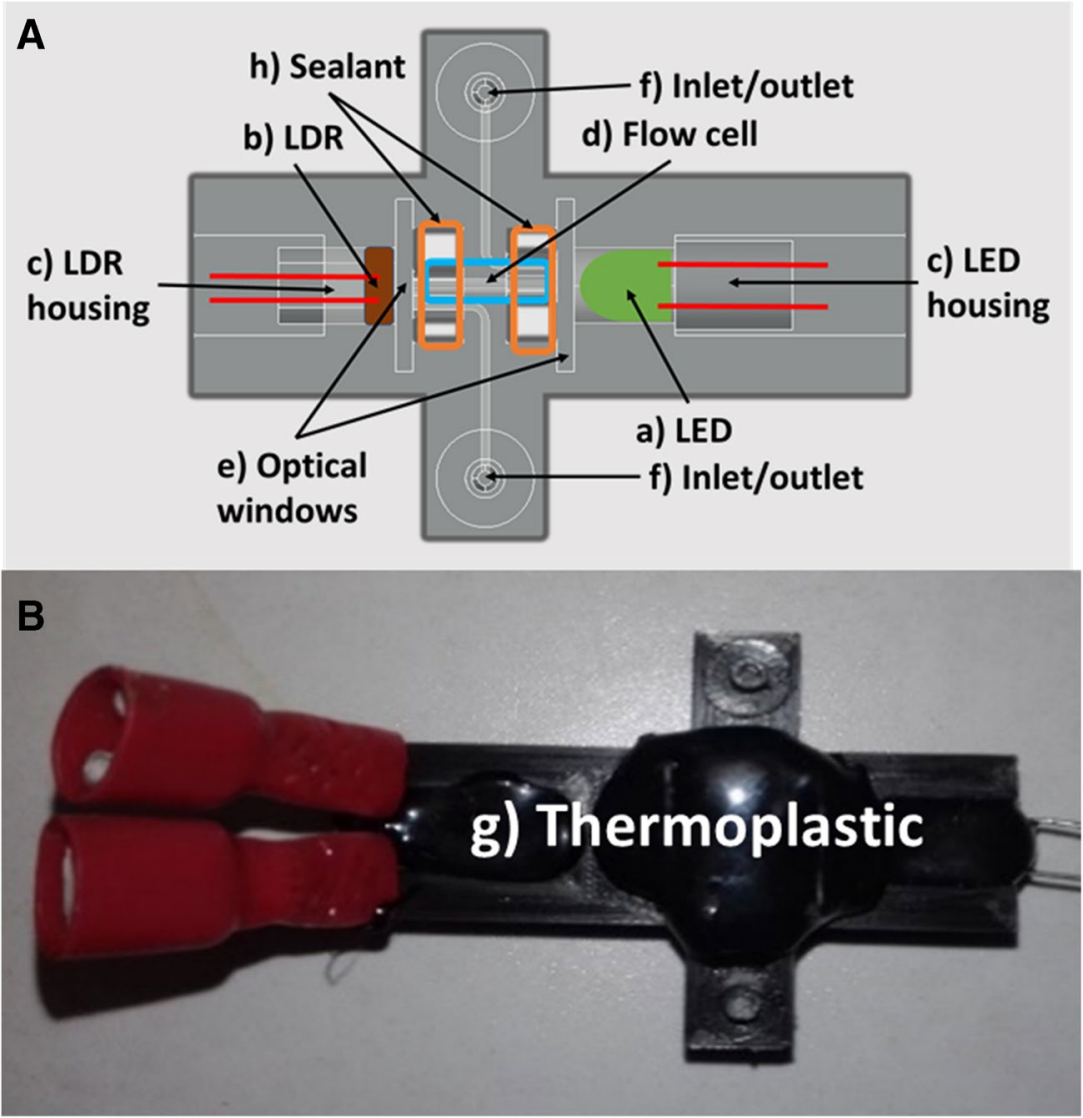
**A** CAD design of the detector flow cell and its different components. **B** 3D-printed photometric detector flow cell assembled

Figure 2A shows a scheme of the 3D-optical flow cell detector designed and fabricated in the work presented herein. Black PLA was chosen to isolate the optical cell and prevent interference from ambient light in the colorimetric measurement of the chelate. The key elements are (a) LED; (b) LDR; (c) the holder for the LED and LDR (Fig. S2A and S2B, respectively); (d) the flow cell which is the channel where light interacts with sample (e) transparent windows; (f) inlets and outlets of sample. Figure 2B is an image of the assembled 3D-printed photometric detector flow cell with a layer of thermoplastic (g) to minimize stray light and ensure optimal optical performance and to prevent vibration of the diodes.

The LED and LDR were mounted in their designated holder within the detector (Fig. S2A and S2B, respectively) whose shape was optimized for the components. To avoid water leaks in the contact between optical windows and flow cell, a sealant NURAL28 (Henckel Ibérica, Barcelona, Spain) (Fig. 2Ah) was used. In addition, a black thermoplastic from SALKI (Barcelona, Spain) was used to fix all components and avoid external light interference.

Regarding the flow cell, both ends were open to allow light from the LED (Fig. S2A) to pass through it and reach the LDR (Fig. S2B). The optical windows (Fig. 2Ae) made of glass (26 mm) closed the microchannel and a sealant was used to block water flow between the contact surface of the window and the flow cell. During the printing process, it was imperative to suspend the ends of the flow cell in the air; however, supports were necessary to achieve a good print, as depicted in Fig. S2C. Once the printing process was complete, the supports were removed.

The detector converts light intensity into a proportional electrical signal (mV) and the signal was collected every 0.1 s. The detector consisted of a green LED (525 nm) and an LDR of 5 mm diameter, and the LED was powered by 3 V and 20 mA. The LDR was integrated with an Arduino UNO for data acquisition purposes, whereby the LDR was connected to a voltage divider circuit. Specifically, the LDR was linked to the second resistance of the circuit, while being directly connected to ground. The first resistance in the voltage divider was 10 KΩ. Thus, the measured voltage was directly proportional to the absorbed light, and therefore to the analyte concentration. Each 100 ms Arduino measured the voltage and sent it to the computer. The CAD design of the detector is illustrated in Fig. S3.

### MicroFIA procedure for Fe(III) determination

The analysis cycle worked as follows. A peristaltic pump (Fig. 1Aa) automatically propelled sample (or standard solution), the carrier (H_2_SO_4_ solution at pH = 2.0) and the chelating reagent into the system. Automatic injection of the sample (25 µL) into the diluter (Fig. 1Ac) was performed by a six-way valve (Fig. 1Ab). As Fe(III) concentration of samples from the bio-reactors could easily reach 6000 mg·L^−1^, the diluter was intended to adjust the Fe(III) concentrations until measurable operational range. The diluted sample was then propelled into the mixer module (Fig. 1Ad) where it came into contact with the chelating reagent (0.180 mg salicylic acid/100 mL) developing a purple color whose intensity was proportional to Fe(III) concentration. After that, the mixture flowed through the disperser (Fig. 1Ae) and finally entered the 3D-optical flow cell (Fig. 1Af), where absorbance was measured and transformed into electrical signals (peak height, mV). Subsequently, the solution was released from the system and discarded as waste. To clean the detector after each measure, a 3-way isolated solenoid valve (Fig. 1Ag), controlled by cooldrive, and a peristaltic pump were used to pump ethanol (Fig. 1Ah). The sequence you can see in Fig. 1B. The total analysis time for a sample is 10.1 min, which means that almost 6 samples can be analyzed per hour.

A software application was developed for the purpose of controlling the system, utilizing LabVIEW 2021. This software was designed to manage the operation of the 6-way valve, facilitated through the implementation of serial communication. Additionally, to enable communication with the Arduino UNO, a package named LINX was installed into LabVIEW. The Arduino UNO was connected to a relay shield 3.0 for Arduino from seed studio, which was utilized to send signals to the pumps and a 3-way isolated solenoid valve. The details of the software specially designed for the control of the analyzer and the active elements of the system (pumps, valves, and data reading/acquisition) are detailed in the Electronic Supplementary Information.

### Spiked and real samples

Several real samples were analyzed to validate the analyzer: medium from a bioleaching reactor, water from a nearby reservoir (Parc de l’Agulla, Manresa, Spain), a mineral medium of a bioreactor without Fe(III) and a leachate obtained from an acid digestion of a battery cathode from a mobile phone. Water from the reservoir was filtered using syringe filters 0.2 μm (Chemlab group, Terrassa, Spain). All these samples were analyzed after adding a known concentration of Fe(III) except the medium from a bioleaching reactor which contains Fe(III) itself.

For accuracy studies, the samples from the bioleaching reactor were diluted with the acidic solution with dilution factors of 1/10, 1/5, 1/2 and 3/4. For each dilution, 2 aliquots were taken, one of them was directly analyzed and in the other, 1500 mg·L^-1^ Fe(III), were added and then analyzed. All the samples were analyzed in triplicate.

Results from microFIA were compared to those obtained with the standard conventional method using a spectrophotometer UV–Vis (PerkinElmer, Lambda25).

### Interference study

An interference study of Cu(II), Al(III) and Fe(II) has been conducted in order to corroborate that there are no interferences caused by these ions that are frequently present in our samples. For this purpose, four aliquots of 22.5 mg·L^−1^ of Fe(III) solutions were prepared. Three of them were spiked with Cu(II) reaching a final concentration of 500 mg·L^−1^ of Cu (solution prepared from CuSO_4_·5H_2_O), 500 mg·L^-1 ^of Al(III) (from Al_2_(SO_4_)_3_·18H_2_O) and 100 mg·L^−1^ Fe(II) (from FeSO_4_·7H_2_O), respectively. The absorbances at 525 nm of Fe(III) solution and the spiked ones were measured by the salicylic colorimetric method at pH 2.0. Moreover, solutions containing only 500 mg·L^−1^ of Cu, Al and 100 mg·L^−1^ Fe(II) have been also analyzed by the same colorimetry. In the Fe(II) solution, 10% (w/v) hydroxylamine was added in order to prevent the oxidation of Fe(II) into Fe(III). The assay was carried out in triplicate.

## Results and discussion

### Optimization of microFIA passive mixing elements

In order to optimize the performance of microFIA device described in the experimental section, multiple operational conditions were tested to ensure optimal system efficiency. The primary focus of the optimization process was to obtain a wide response range of Fe(III) concentrations, improve the degree of mixing within the analyzer, and enhance detector stability.

#### Diluter

The diluter served to adjust the sample concentration to ensure compatibility with the operational range response of the micro flow injection analysis system. Given that samples sourced from the bioleaching reactor were anticipated to exhibit high Fe(III) concentrations, potentially reaching up to 6000 mg·L^−1^, this step was crucial. The diameters of the inlets, outlets, and channels within the diluter were of utmost significance, as they played a pivotal role in facilitating the proper flow of the solvent and sample mixture.

To optimize the dilution process and minimize overpressure on the diluter module, dilution was performed at three distinct points. The main channel of the diluter comprised two serpentine sections, followed by a straight section. The internal width of the main channel was progressively increased by 0.5 mm in each successive section. The first section measured 43 mm in length and 1 mm in width, the second section measured 67 mm in length and 1.5 mm in width, while the final section measured 17 mm in length and 2 mm in width. This design helped to facilitate the mixing process and reduce the pressure on the diluter. Each inlet was connected to a rectangular channel measuring 0.5 mm in width, 0.5 mm in height, and 4 mm in length. The optimal diameters of the inlet points were 1 mm, while the outlet point was 1.5 mm. This configuration was found to yield the best results. The CAD design of the mixer reactor is illustrated in Fig. S4.

#### Mixer

In the mixer or reactor, the diluted sample underwent a reaction with the chelating reagent, resulting in the formation of a purple chelate that exhibited absorbance at 525 nm. The intensity of the signal was directly proportional to the concentration of Fe(III) present in the sample. The CAD design of the mixer reactor is illustrated in Fig. S5. The reactor comprised three separate entrances, with one dedicated to the introduction of the diluted sample, and the remaining two reserved for the introduction of reagents, serving to increase the reagent ratio. The reactor channel was designed with a succession of linear segments and square turns, which served to improve mixing efficiency [38], culminating in the outlet channel.

The width and height of the channels within the microfluidic system were critical to ensure efficient and reliable performance. Channels with a height of 1 mm were used to prevent overpressure and liquid leaks from occurring at the connections. The sample microchannel had a width of 0.5 mm, and connected to the reagent channel at a T-junction, which had a width of 1.0 mm. A second reagent inlet, also with a width of 1.0 mm, joined the main channel, resulting in a final channel width of 2.0 mm. The large mixing zone within the mixer (as shown in Fig. S5) provided ample time for the sample and reagent to react completely and allowed for an initial dispersion of the chelate obtained to reduce peak height and prevent detector signal saturation. To ensure optimal mixing efficiency, the mixer featured 76 90-degree turns, with each turn measuring 24 mm in length and 5.3 mm in width, which generated sufficient turbulence within the channel.

#### Disperser

The addition of the disperser module was a key improvement to the microFIA design, as it allowed for better dispersion of the chelate, reducing the peak height and avoiding the detector signal saturation. The 3D-platform disperser was designed to be placed after the mixing module and before the 3D-optical microflow cell. It consisted of a rectangular serpentine-shaped main channel with smaller rectangular serpentine segments to increase turbulence and improve chelate dispersion. The inlet diameter was 1.5 mm, the outlet was 2 mm, and the channel dimensions were 2 mm width and 1 mm height, all of which helped to minimize overpressure in the output. Fig. S6 shows the design of the disperser module in detail.

### Optimization of detector response

Based on the results, it was found that the best color to print the detector was black PLA as it provided the highest voltage difference between the red dye solution and water, indicating minimal external light interference. Additionally, the dimensions of the flow cell were optimized to ensure a good fit of the LED and LDR orientation, resulting in improved detector performance.

Table S1 shows that the average voltage difference between water and dye solution was lower for the transparent and white flow-cells, indicating higher interference due to external light. The black flow-cell showed the highest voltage difference, indicating the least interference due to external light. This was because the black PLA absorbed all external light and only allowed the light from the flow-cell to reach the LDR. Therefore, the black flow-cell was chosen as the optimal color for printing the detector.

The baseline was the signal obtained when only the solvent or reagent flowed through the detector without any sample. By measuring the baseline, any fluctuation or drift in the detector could be accounted for and eliminated, resulting in more accurate measurements. In this case, measuring the baseline for 20 s before the chelate measurement helped to stabilize the baseline and improve the repeatability of the measurements. The analytical signal was then calculated as the difference between the signal of the chelate and the signal of the baseline. This approach helped to minimize any interference or noise in the measurement and ensure more accurate and reliable results.

### Optimization of operating conditions

After obtaining the baseline signal, the diluted sample was introduced into the system and the analyte was analyzed by measuring the signal of the chelate formed between Fe(III) and salicylic acid. The intensity of the signal was proportional to the Fe(III) concentration in the sample and the signal was measured by the LDR in the 3D-optical micro flow-cell and processed by the Arduino microcontroller. The output signal was then sent to a computer for data analysis.

After that, the injection valve (Fig. 1Ab), in load position, was filled (25 µL) with the sample or standard solution and introduced in the system by the carrier. The flow rate of different solutions was optimized after several assays and was finally established as follows: carrier 0.33 mL/min; diluent 1, 0.40 mL/min; diluent 2, 0.37 mL/min; diluent 3, 0.39 mL/min; reagent 1, 0.41 mL/min; and reagent 2, 0.32 mL/min. After each measurement, the detector was washed with ethanol that was introduced in the disperser with a second pump and a 3-way-valve (Fig. 1Ag). The flow rate of ethanol was 1.36 mL/min. Total analysis and cleaning time was 10.1 min (Fig. 1B). To avoid the formation of bubbles that would interfere in the measure, all solutions were previously sonicated.

### Validation of microanalyzer performance

In order to prove the microanalyzer’s suitability, reliability and robustness for the continuous monitoring of Fe(III) in a bioleaching reactor, the microFIA system was validated. The parameters determined for this purpose were precision (repeatability and reproducibility), limit of detection and limit of quantification, linear response range, accuracy and real sample applications.

#### Precision

##### Repeatability

The precision expressed as repeatability was determined by analyzing standard solutions with different concentrations of Fe(III) several times. The closeness between the multiple obtained results indicates the repeatability of the method. The precision study was performed in a wide range of Fe(III) concentrations (150, 1500, 3000, 4500 and 6000 mg·L^−1^). Every concentration was analyzed in three different days in triplicate (n = 9). For each concentration, the mean, standard deviation and the experimental coefficient of variation (CV) were calculated. The experimental CV obtained was compared to Horwitz coefficient (C_vh_) [39]. The C_vh_ = 2^(1–0.5·log(C))^, where c is the concentration of analyte in g/mL. When experimental CV < C_vh_, it means the precision is acceptable. Table S2 shows analytical results for each concentration, the average, standard deviation, and coefficient of variation as well as the coefficient of Horwitz.

**Fig. 3:**
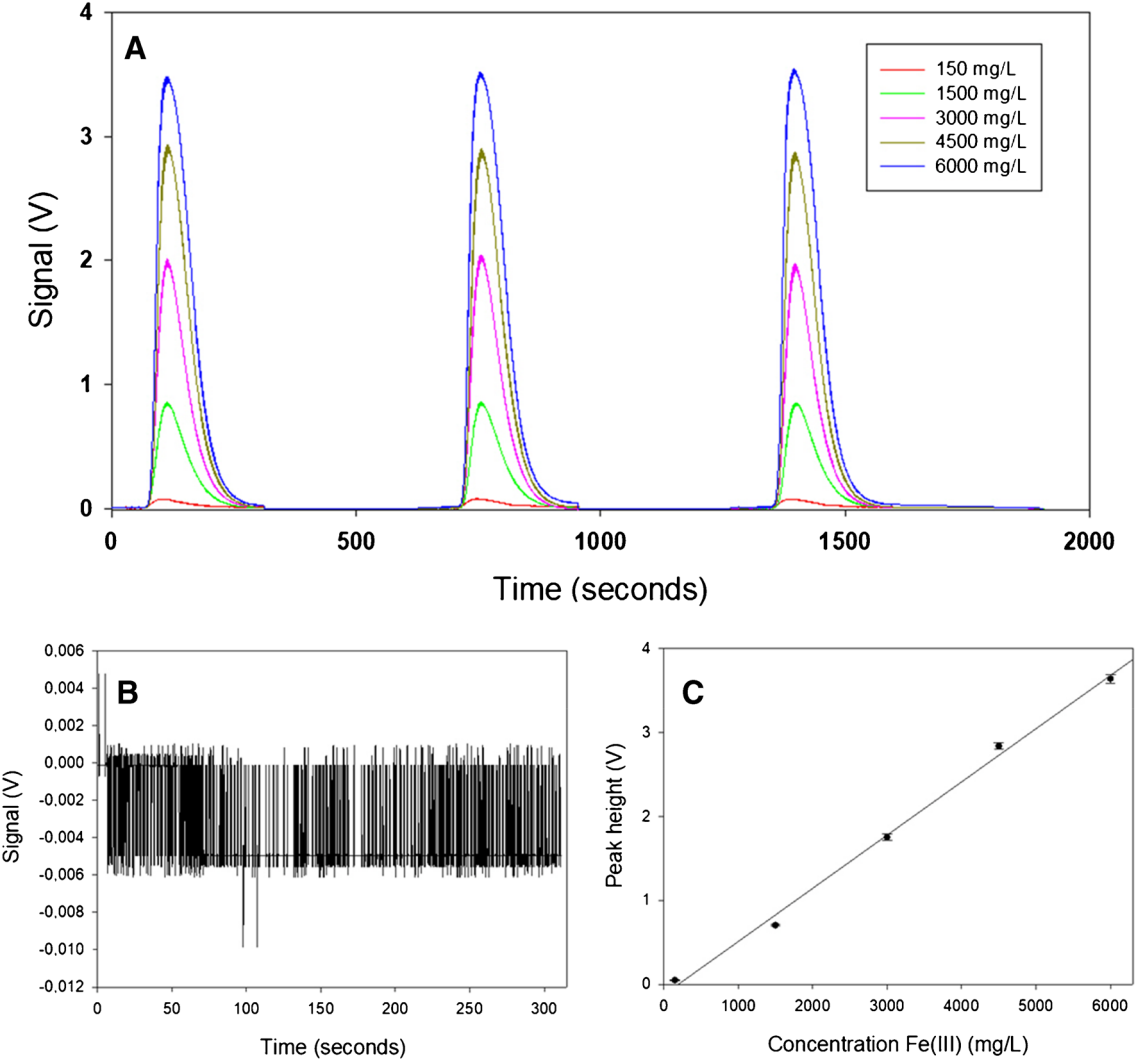
Calibration performed in the microFIA at optimal conditions. (**A**) Analytical signal (λ = 525 nm) at different concentrations, (**B**) blank and (**C**) calibration plot. The equation of the calibration curve is Voltage = 0.00063 (± 0.00002) [Fe(III)]—0.12 (± 0.09) (*r*^2^ = 0.996; *n*=5 in triplicate). Operational conditions are injection volume 25 µL, carrier flow rate 0.33 mL/min, diluent flow rate 0.39 mL/min, reagent flow rate 0.36 mL/min, and the ethanol flow rate 1.36 mL/min

As Table S2 shows, the CV was smaller than 2% for almost all concentrations of Fe(III), except for the smallest one of 150 mg·L^−1^. In any case, the experimental coefficients were significantly lower than the maximum acceptable value given by Horwitz for each concentration. Thus, the response of microanalyzer in the range of concentration between 150 and 6000 mg·L^−1^ of Fe(III) was repeatable. Figure 3 shows the peaks of the calibration points (Fig. 3A), blank (Fig. 3B), and calibration plot (Fig. 3C).

##### Reproducibility

In this study, the reproducibility was studied in terms of the variation of the sensitivity of the system over time. Sensitivity is represented by the slope of the calibration curve. Thus, the calibration of the microFIA system on successive days (*n* = 7) under the optimal operational conditions was studied. With an average sensitivity of 0.000634 V L/mg and a standard deviation of 0.000020 V L/mg, the response of the microFIA in successive calibrations was reproducible (Table S3). An acceptable relative standard deviation (RSD) of sensitivity of 3.1% (less than 5%) was obtained.

#### Limit of detection and limit of quantification

The limit of detection (LoD) and limit of quantification (LoQ) were determined by signal-to-noise approach, a method suitable for analytical procedures that exhibit baseline noise. The signal-to-noise ratio was carried out by blank analysis (Fig. 3B). To determine the LoD and LoQ, 278 measurements of baseline from blanks were performed. The mean and standard deviation values were then calculated according to [40]: LoD = (X + 3*Sb)/S and LoQ = (X + 10*Sb)/S, where Sb is the standard deviation of the blank mean and S is the slope of the calibration curve or sensitivity. From this method, the limit of detection was 11 mg·L^−1^ and the limit of quantification was 25 mg·L^−1^.

#### Linear response range

The linearity of an analytical method expresses the relationship between analyte concentration in the sample and the analytical signal obtained (in this case, height peak in volts). The linear response range of an analytical method is the interval between the upper (limit superior of quantification) and LoQ. In this study, linearity of the designed analyzer was determined by a series of 3 analyses of 5 different Fe(III) standard solutions whose concentrations were between 25 and 6000 mg·L^−1^. From the obtained results, the calibration plot by the method of least squares was calculated. The *r*^2^ was 0.996 provided that pH was under 2.0. The calibration plot is shown at Fig. 3C. This linearity range is very wide, higher than those described in the literature [29, 32–36].

#### Accuracy

Accuracy, which indicates the degree of agreement between the experimental data and the true value, can be expressed as recovery (%) of an added standard in the sample. Here, recovery was determined by spiking a sample from a bioleaching reactor containing different Fe(III) concentrations (150 to 6000 mg·L^-1^) with a standard solution of 1500 mg·L^−1^ Fe(III). All samples were analyzed 3 times before and after being spiked.

The recovery percentage was calculated as % R = (|Y -X|/1500)·100, being X the Fe(III) concentration before spiking and Y after standard addition of 1500 mg·L^−1^. Results for the microFIA analyzer are shown in Table 1.

**Table 1:** Accuracy results of samples from bioleaching reactor using microFIA system

	Samples		Spiked samples		
Sample	Average (mg·L^-1^)	SD	Average (mg·L^-1^)	SD	Recovery (%)
Sample 1	636	8	2115	60	98.64%
Sample 2	1296	19	2806	90	100.67%
Sample 3	3157	11	4673	30	101.08%
Sample 4	4517	50	6012	8	99.46%

As can be seen in Table 1, the microFIA method developed in the present work is accurate in the range concentrations between 500 and 6000 mg·L^−1^. A recovery of around 100% in the analyzed samples was obtained. The wide range of concentrations in which it can be applied and the additional advantage of automatically performing the dilution of the sample are noteworthy.

#### Interference study

The determination of Fe(III) by colorimetry using salicylic acid as a complexing agent may experience some interference. According to [41], some anions (arsenate, citrate, dichromate, cyanide, thiocyanate, orto- and pyrophosphate, fluoride, iodide, tungstate) and metallic ions (Al(III), Cu(II), Ba(II), Be(II), Bi(III), Cr(VI), Co(II), Mn(II), Hg(I), Sn(II), Sn(IV) and Th(IV)) can interfere the determination of Fe(III) with salicylic. In the samples for which the analyzer has been designed (bioleachates from PCBs), these chemical species that could cause interference are not found, with the exception of Al(III), Cu(II) and Fe(II). However, it should be noted [41] that the interferences occur at a pH greater than 2.5. According to some authors, these ions don’t cause interferences in the determination of Fe(III) with salicylic acid at a very low pH = 1.8–2.0 that are precisely our conditions [42, 43].

Also it has been demonstrated that Hg^2+^, Fe^2+^, Pb^2+^, Cd^2+^, Na^+^, K^+^, Zn^2+^, Ca^2+^, Mg^2+^, Sn^2+^, NO_3_^−^, NO_2_^−^, CO_3_^2−^, SCN^−^, oxalate, and citrates do not interfere with Fe(III)-salicylic under high acidic conditions [44].

Regarding Fe(II) interference, according to [37], 100 mg·L^−1^ of Fe(II) also does not interfere in the determination of Fe(III) with salicylic acid as a chelant under acidic conditions. In fact, to determine Fe(II) and Fe(III) simultaneously using the sulfosalicylic acid, Fe(III) is first determined under acidic conditions, and then the medium is made alkaline, and under these alkaline conditions, total iron (Fe(II) + Fe (III)) is then determined [18].

**Fig. 4:**
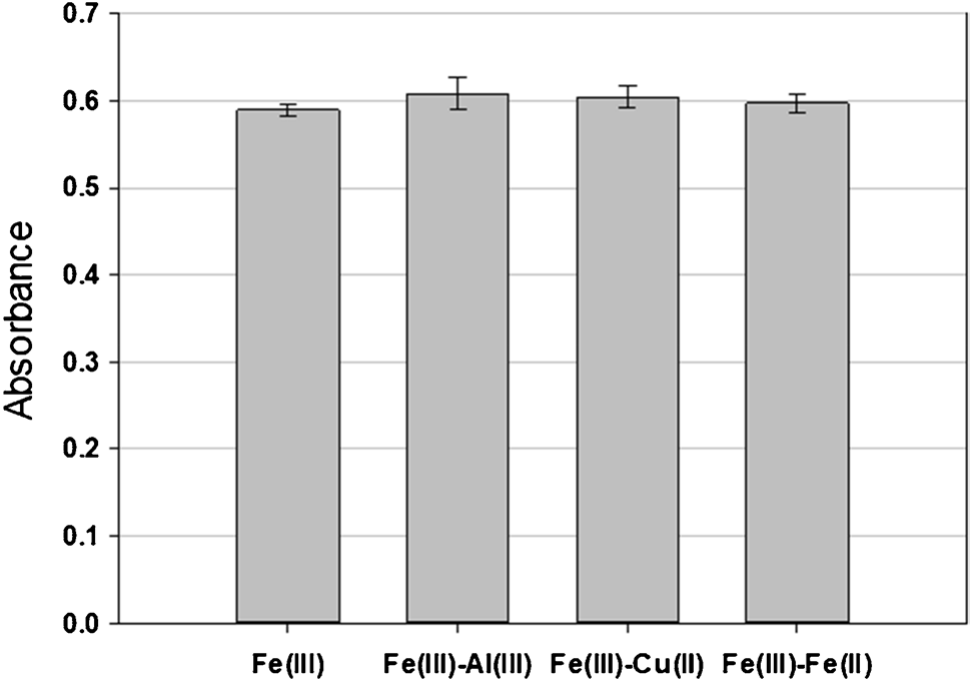
Interference study. Comparative absorbance (λ = 525 nm) for Fe(III) and mixtures of Fe(III) and potentially interfering ions

In the present study, the potential interference of Cu(II), Al(III), and Fe(II) ions in the determination of Fe(III) was investigated. The results are displayed in Fig. 4. As it can be seen, Cu(II), Al(III) and Fe(II) did not produce interference in the determination of Fe(III) under our working conditions (pH = 1.8–2.0), even at concentrations of 500 mg·L^−1^ for Cu(II) and Al(III), and 100 mg·L^−1^ for Fe(II). On the other hand, the absorbances shown in the Table S4, indicate that the absorbance of these potential interferent ions using salicylic acid as a chelant is negligible under acidic conditions.

The results of the characterization study are presented in Table 2, which summarizes all the quality parameters of the method, including accuracy and precision (repeatability and reproducibility). The sensitivity of the method is suitable for application in bioreactors and allows the wide linear response range from 25 to 6000 mg·L^−1^ Fe(III). On the other hand, the reproducibility of the method, determined as the relative standard deviation of the sensitivities on different operation days (RSD = 3.1%), ensures the proper response of the analyzer for process monitoring.

**Table 2:** Principal parameters of quality of the automatic microFIA system developed for determination of Fe(III)

Parameter	Value
Sensitivity (V L mg^−1^)	0.00063 ± 0.00002
Detection limit (mg·L^−1^)	11
Quantification limit (mg·L^−1^)	25
Linear range (mg·L^−1^)	25–6000
Repeatability (CV) (*n* = 9)	0.69–4.92%
Reproducibility (RSD) *(n *= 7)	3.1%
Selectivity	At pH acids not interference was observed
Sample throughput (h^−1^)	6
Accuracy (RV%)	99.9 ± 1.1

In Table S5, the characteristics of other analytical methods based on different reagents and automation techniques such as FIA [36, 45, 46], SIA [19, 47] and microsystems [11, 32, 33, 35] are shown. The main advantages of our analyzer over other automatic analysis systems are the wide linear response range and the minimization of interferences it presents. The low LoD (11 mg·L^−1^) obtained by our system is also surprising comparable to that of other microFIA systems [35] (20 mg·L^−1^) which have a much narrower working range (40–305 mg·L^−1^) and more interferences such as Cu(II). Kim et al. [11] present a very sensitive microfluidic system based on fluorescence measurements with a very small application range (0–12 mg·L^−1^) but with interference from Al(III). While other FIA systems [36] have a wider dynamic range (110–560 mg·L^−1^), they also have a higher LoD (110 mg·L^−1^) and a high selectivity. It is important to highlight the high accuracy (99.9%) and precision (3.1%) of this analyzer comparable to those of other more sensitive microFIA systems [32, 33, 35]. On the other hand, the high analysis frequency of classical flow systems such as FIA [45] and SIA [47] between 25 and 102 h^−1^, respectively, is not feasible for our system. The high degree of dilution required to extend the linear response range causes the integration of multiple mixing and dilution stages that reduce the analysis frequency to 6 h^−1^.

#### Real sample applications

The microFIA system was evaluated for its applicability by analyzing several real samples with increasing matrix complexity in a final assessment. The first sample was water from the Agulla reservoir in Manresa, Spain, which has a complex composition and is rich in organic matter. The second sample was the growth medium typically used for *Acidithiobacillus ferrooxidans*, the microorganism used in bioleaching process. The third sample was a leachate obtained from acid leaching of the black mass from ion-Li mobile batteries, while the last sample was a bioleachate from the bioleaching reactor, which was the only sample containing naturally occurring Fe(III).

A total of 12 real and spiked samples were analyzed by the developed microFIA system in triplicate for several days. To compare the results, they were also analyzed by a UV–vis conventional method. Table 3 presents the analytical results obtained from both methods. To evaluate the method statistically, a paired-test and least-square linear regression were used, and no significant differences were observed at a 95% confidence level. In the paired test: t_cal_ = 0.91 < t_tab_ = 2.07, while in the least square linear regression the slope and intercept were 1.02 ± 0.02 and 21 ± 43, respectively (*r*^2^ = 0.996; *n* = 12; 95% confidence level).

**Table 3:** Results from the microFIA system of the different samples compared with the results from UV–Vis method

		FIA		UV–Vis	
	[Fe(III)] added (mg·L^−1^)	Average (mg·L^−1^)	SD	Average (mg·L^−1^)	SD
Bioleaching reactor	-	636	8	713.8	1.1
	-	3157	11	2962	70
	-	2115	60	2120	5
Water from lake in “Parc de l’agulla”	300	377	6	249	4
	600	608	8	518.7	0.6
	1000	915	24	834.1	1.6
Mineral medium	1050	1042	11	924.0	0.6
	2000	1941	60	1930	4
	4050	4289	40	4211	13
Leachate from dissolved mobile batteries in acid	500	428	6	402	4
	2500	2267	90	2312	24
	3500	3444	40	3340	6

To showcase the microFIA analyzer’s capability to continuously and automatically monitor Fe(III) concentration in an in situ bioreactor, the system was integrated with a reactor that was oxidizing copper from electrical cables by converting Fe(III) to Fe(II). The microFIA analyzer was able to measure the decreasing Fe(III) concentration in real time, with measurements programmed every 20 min.

**Fig. 5:**
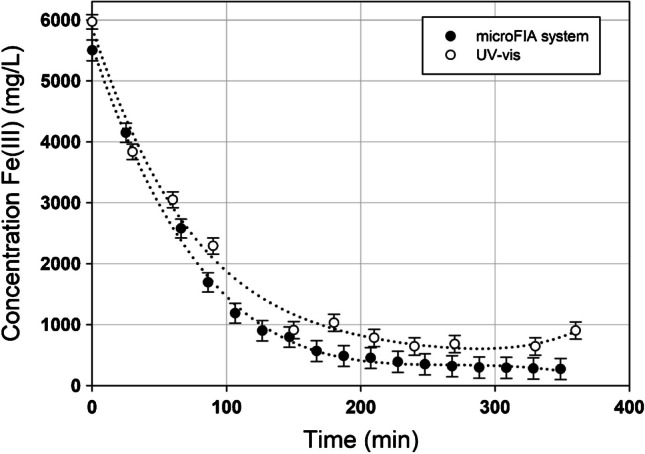
Comparison between online measurement using new microFIA system (*n* = 1) and batch determination UV–Vis method (*n* = 3). Experimental uncertainty is determined for error interpolation

To validate the microFIA analyzer’s suitability for this purpose, the results obtained were compared against those acquired through batch measurements using UV–Vis analysis. The results of this comparison are presented in Fig. 5.

The results illustrate that the microFIA system is capable of on-line Fe(III) monitoring in real-time, without the need for any previous sample pretreatment, with programmed measurements taken automatically every 20 min. Furthermore, the system was able to perform this monitoring smoothly and demonstrated no significant differences when compared to the UV–vis method.

## Conclusions

The microfluidic system designed and manufactured in this work has proven to be a highly suitable instrument for continuously and automatically determining the Fe(III) concentration in a bioreactor in a wide concentration range, from 25 to 6000 mg·L^−1^.

The system was successfully validated in terms of precision (repeatability and reproducibility), limit of detection and limit of quantification, linear response range, accuracy, and real sample applications. Repeatability was less than 5% coefficient of variation throughout the concentration range, and accuracy was around 100%.

The effectiveness of the system was also evaluated for its applicability by analyzing four real samples with different matrix complexities and no significant statistical differences were found compared to a conventional UV–Vis method.

The use of the 3D technology for manufacturing the proposed device allows the creation of different pieces in a quickly, precise, and independent way. The technique is highly adaptable and permits making changes on the fly in an easy and cost-effective manner.

Taken together, the results demonstrate several advantages of combining miniaturization and continuous flow techniques in a symbiotic association to achieve on-line monitoring of high concentrations of Fe(III) in complex sample matrices. These advantages include lower cost due to reduced sample and reagent volumes, less maintenance, lower waste disposal, and lower personnel costs because the system is suitable for unattended operation over extended periods of time.

The FIA technique is an open approach to automate analysis processes. Combining FIA with 3D-printed platforms enables the adaptation of analyses based on the formation of coloured products. The determination of other metals or ions of interest by the formation of coloured complexes can be implemented by modifying the wavelength of the LED in the flow cell and some characteristics of the modules. This study provides a novel strategy for future work, making it easier to extend to other target analytes based on colorimetric reactions.

### Supplementary Information

Below is the link to the electronic supplementary material.

Supplementary file1 (PDF 1.60 mb)

## Data Availability

The data that support the findings of this study are available from the corresponding author upon reasonable request.
