# Supplementary material for: Microflow injection analysis based on modular 3D platforms and colorimetric detection for Fe(III) monitoring in a wide concentration range
Source: Mikrochim Acta. 2023 Dec 2;191(1):3. doi: 10.1007/s00604-023-06029-x (PMC10693521; doi:10.1007/s00604-023-06029-x)
Supplement: Supplementary file 1 — Supplementary file1 (PDF 1.60 mb) [file 604_2023_6029_MOESM1_ESM.pdf]

Supplementary Information

Microflow injection analysis based on modular 3D platforms and colorimetric detection for Fe(III) monitoring in a wide concentration range

David Ricart[1], Antonio David Dorado[1], Conxita Lao-Luque[1], Mireia Baeza[2,*]

[1]*Universitat Politècnica de Catalunya, Avinguda de les Bases de Manresa 61-73, 08240 Manresa, Spain*

[2]*GENOCOV Research Group, Department of Chemistry, Faculty of Science, Edifici C-Nord, Universitat Autònoma de Barcelona, Carrer dels Til·lers, 08193 Bellaterra, Spain*

* Corresponding author: mariadelmar.baeza@uab.cat

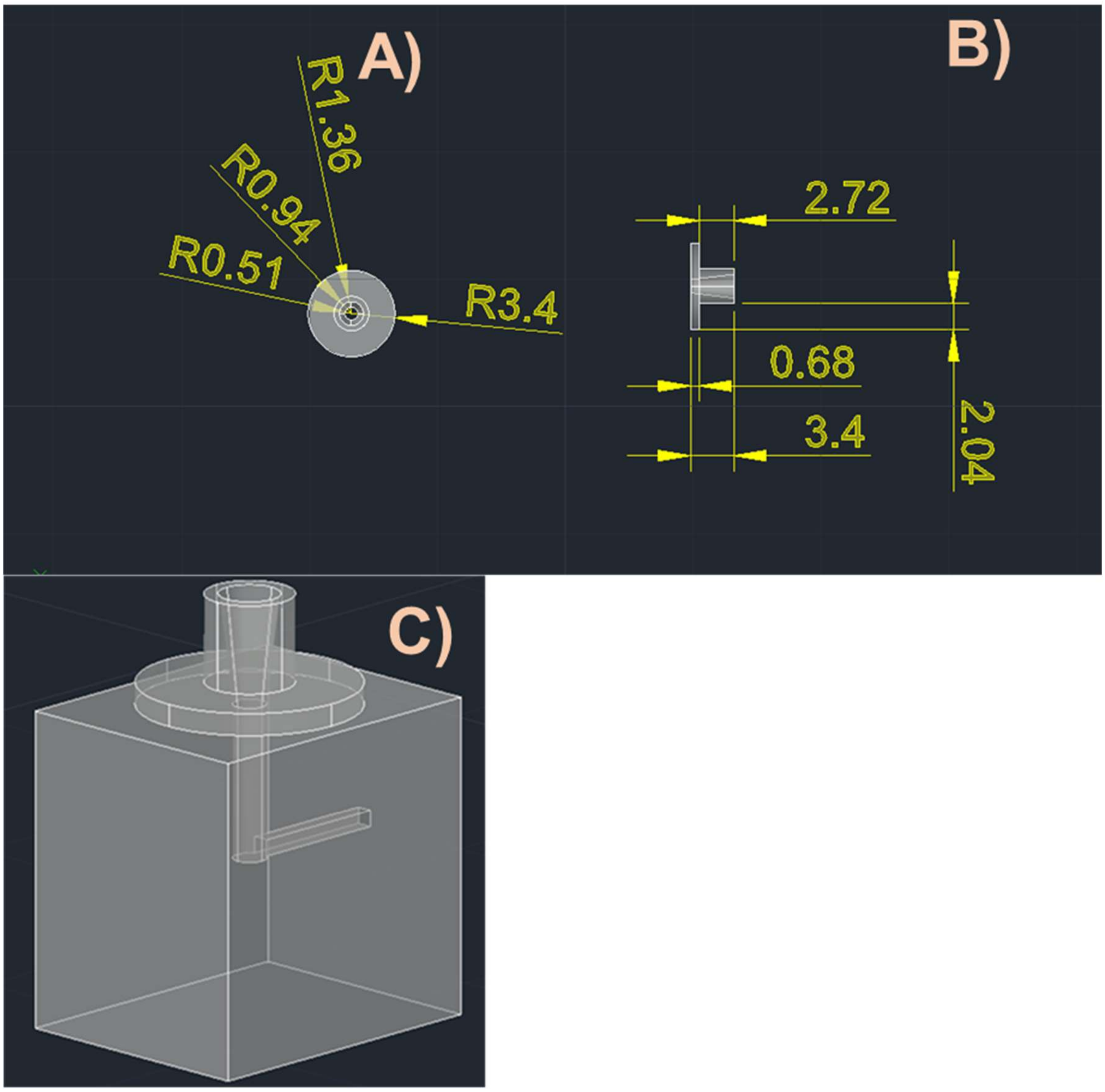

Figure S1. CAD of the inlet/outlet of the modules. A) Top view, B) side view, and C) connection with the module. The units for dimensions are in millimeters.

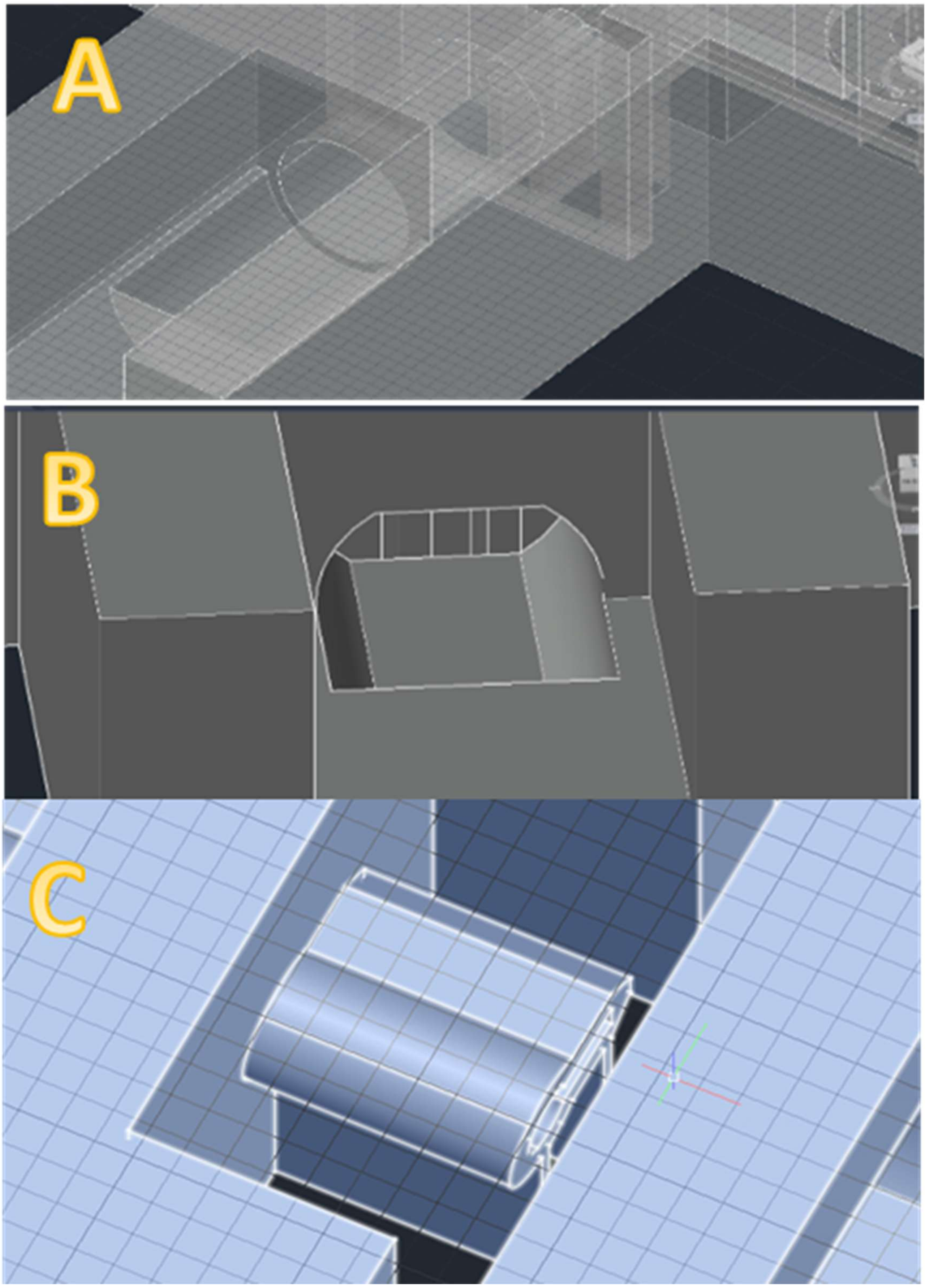

Figure S2. A) Housing LED. B) Housing LDR. C) CAD design of suspended microchannel with its supports.

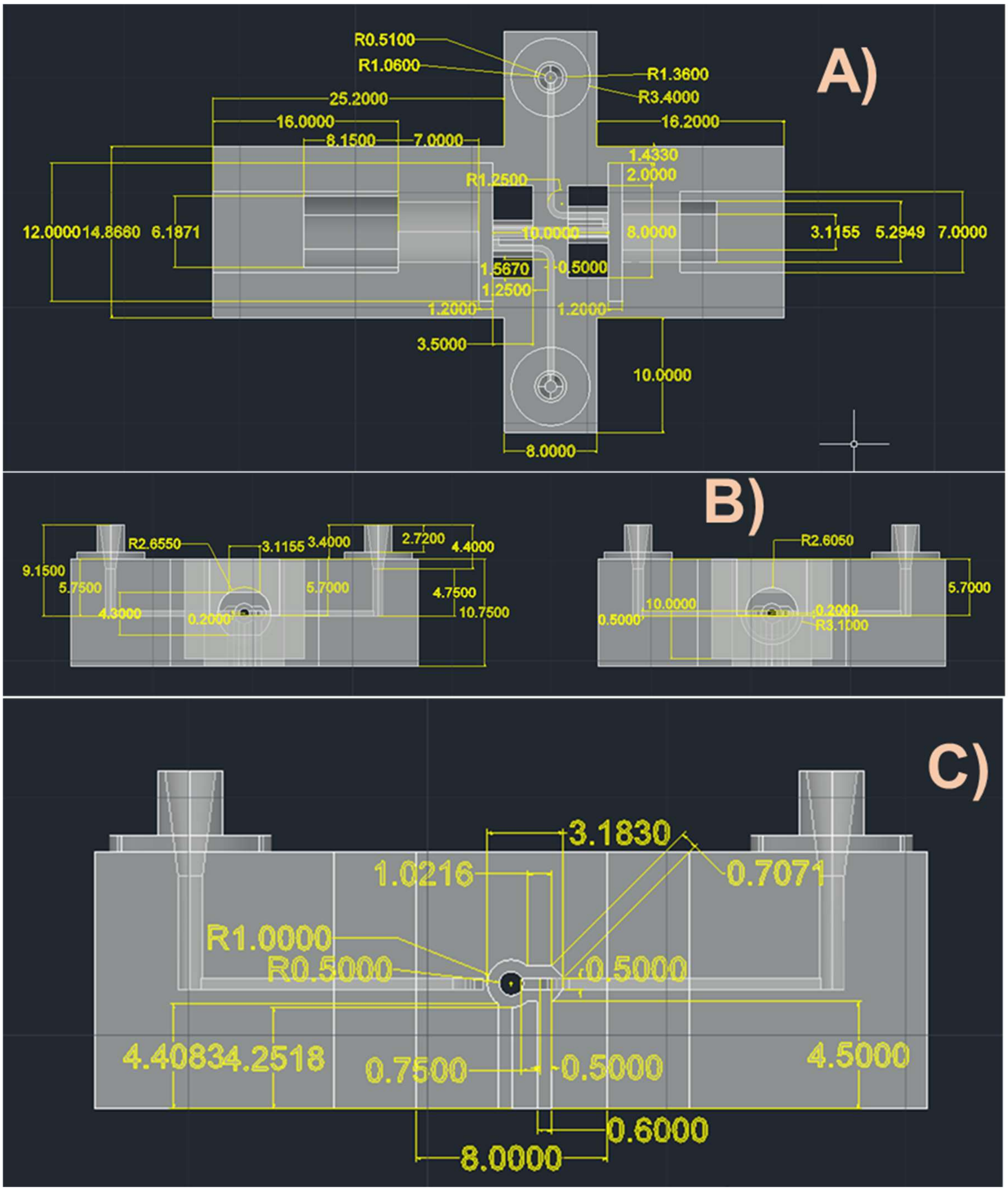

Figure S3. Dimensional labeling of the detector in CAD. A) Top view, B) side view, left is the LDR housing and right the LED housing, and C) flow cell view. The units for dimensions are in millimeters.

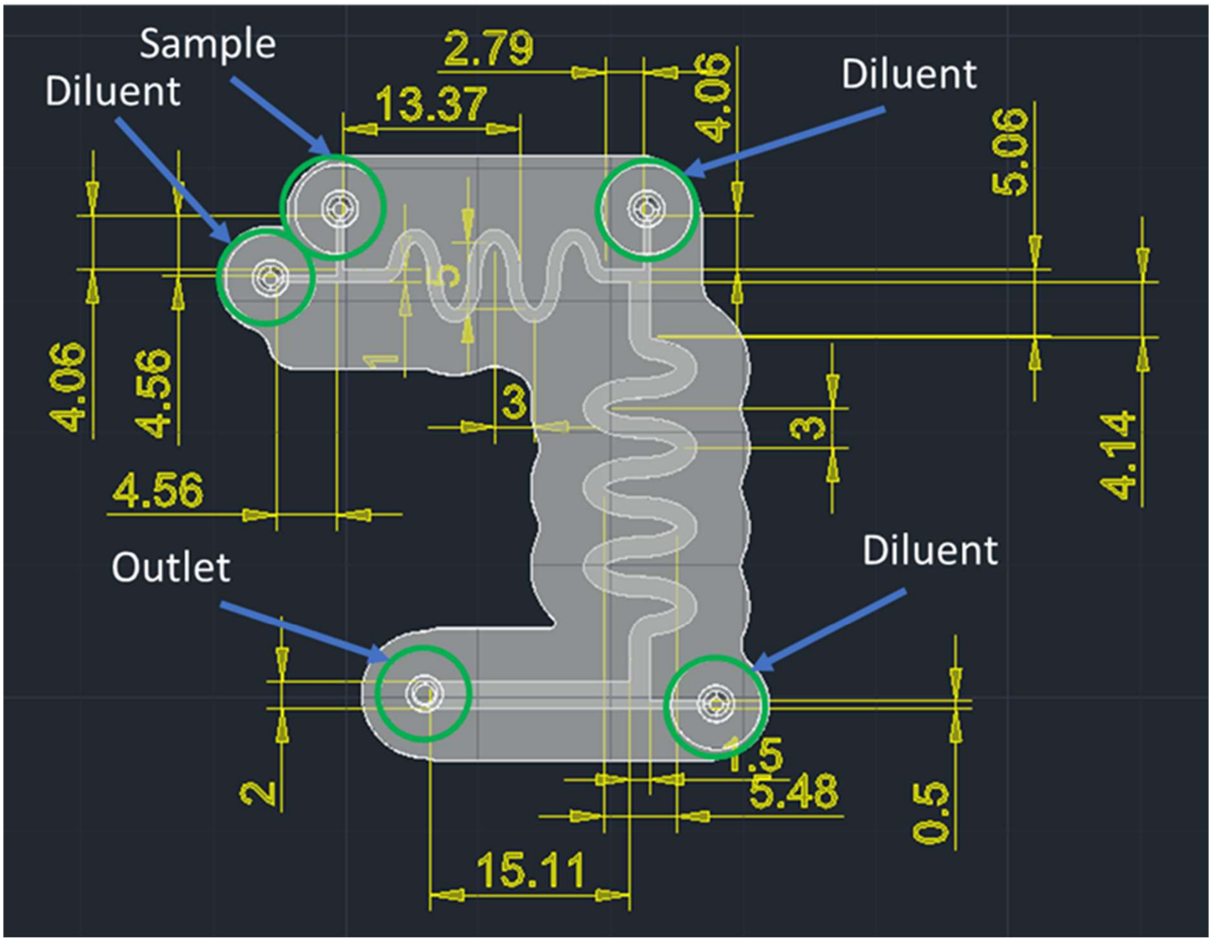

Figure S4. Dimensional labeling of the diluter in CAD. The units for dimensions are in millimeters.

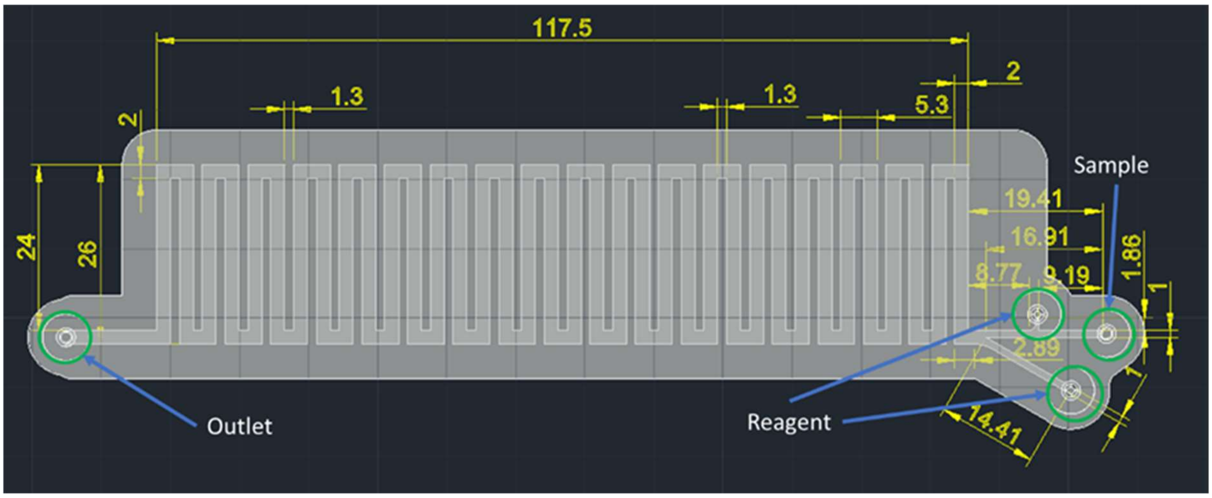

Figure S5. Dimensional labeling of the mixer in CAD. The units for dimensions are in millimeters.

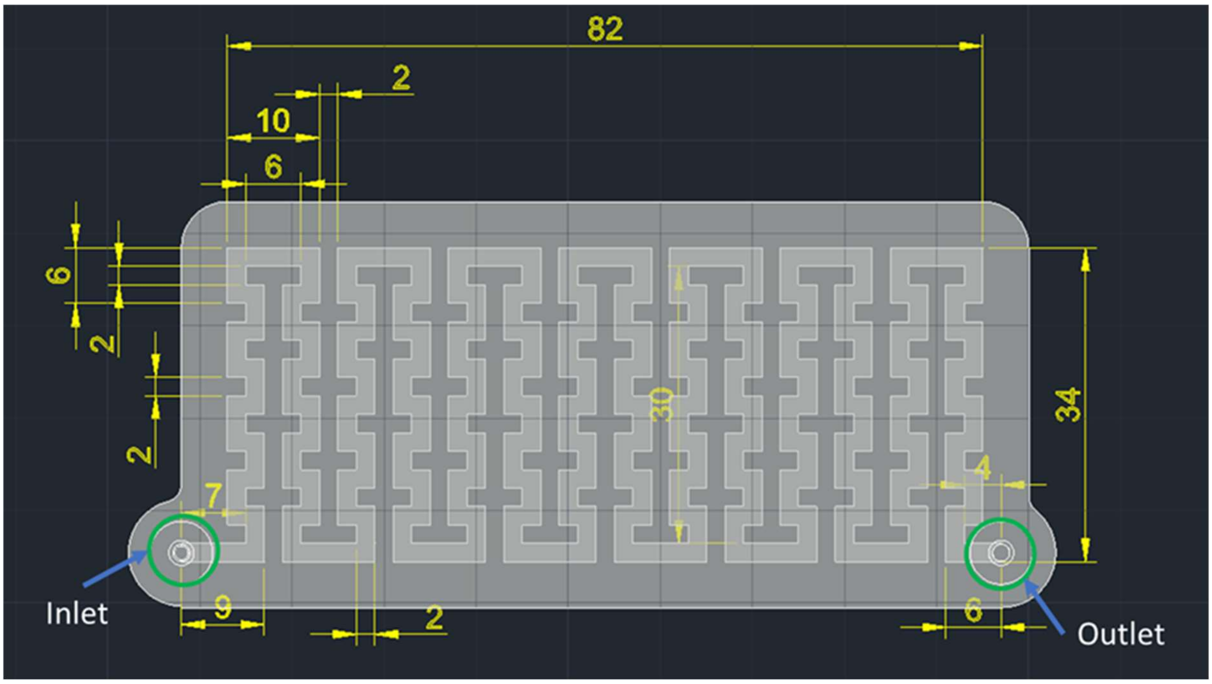

Figure S6. Dimensional labeling of the disperser in CAD. The units for dimensions are in millimeters.

Details of the software specially designed for the control of the analyzer

The system was mounted as follow. The 6-way valve was connected via serial communication RS-232 to the PC. Arduino UNO communicates with PC via USB. LabVIEW is the software that controls Arduino and receives information from it. The Arduino PINs to read or write signals are defined in the LabVIEW interface. The LED was controlled by a digital signal to turn on or off. The Arduino UNO PIN that emits 5 V is used to power the LDR. A voltage divider circuit is used to convert the change in resistance of the LDR to a change in voltage, which is read as an analog input by the Arduino UNO. A relay module on top of the Arduino to control the pumps since the wires connection with the current was cut to be controlled with a relay. The NResearch solenoid valve was controlled by the Cool Drive Valve 161D5X12 of NResearch, which was powered by an electric transformer. The signal to change the position of the solenoid valve comes from a digital PIN on of Arduino UNO, which receives the Cool Drive 161D5X12.

Table S1. Average voltage of 200 measures to test different PLA colors.

	Transparent PLA		White PLA		Black PLA	
Solution	**Water**	**Dye**	**Water**	**Dye**	**Water**	**Dye**
Average (V)	0.68	1.44	1.23	2.81	1.26	3.95
Difference(V)	0.76		1.58		2.68	

Table S2. Results of repeatability of the modular microFIA system (n=9).

**Concentration Fe(III) (mg L^{-1})**	**Average (V)**	**σ^2**	**CV**	**C_{vh}**	**$CV<C_{vh}$?**
**150**	0.072	0.004	4.92%	7.53%	Acceptable
**1500**	0.861	0.006	0.69%	5.32%	Acceptable
**3000**	2.04	0.04	1.81%	4.79%	Acceptable
**4500**	2.92	0.04	1.48%	4.51%	Acceptable
**6000**	3.51	0.05	1.36%	4.32%	Acceptable

Table S3. Reproducibility study of microFIA system (n=7).

	Sensitivity ($V \cdot L \cdot mg^{-1}$)	y-intercept (V)
	0.00066	-0.04
	0.00066	-0.06
	0.00064	-0.09
	0.00063	-0.09
	0.00062	-0.09
	0.00060	-0.18
	0.00063	-0.12
**Average**	**0.000634**	**-0.10**
**SD**	**0.000020**	**0.04**

Table S4. Interference study. Absorbance at 525 nm of potentially interfering ions analyzed with the manual method individually.

Sample	Average (Abs)	Standard deviation (Abs)
**Al(III)**	0.014	0.006
**Cu(II)**	0.008	0.013
**Fe(II)**	0.016	0.007

Table S5. An overview on better reported optical methods for the determination of Fe(III).

Method	Linear range	Material of channels	Reactant	Precision (RSD%)	Accuracy (RV%)	Selectivity	Measure frequency (h^{-1})	LoD	LoQ	Reference
Fluorescence spectra	0-12 $mg\cdot L^{-1}$	SU-8 photoresist	Ethylenediamine derivative of a rhodamine 6G silica particle	-	-	High, only affects Al(III)	-	-	-	[11]
SIA	0.05-3 $mg\cdot L^{-1}$	Tygon pump tubing, PTFE tubing	Deferiprone	<5	96 - 104	High, (200 $mg\cdot L^{-1}$ Zn^{2+}, Cd^{2+} and Ca^{2+}) (3 $mg\cdot L^{-1}$ of Al^{3+})	60	0.032 $mg\cdot L^{-1}$	0.055 $mg\cdot L^{-1}$	[19]
µFA	0.05-4 $mg\cdot L^{-1}$	PMMA	Nitroso-R salt	<2	98.7±0.12	Low	40	0.021 $mg\cdot L^{-1}$	0,081 $mg\cdot L^{-1}$	[32]
µFA	0.2-5 $mg\cdot L^{-1}$	PMMA, sealed with PDMS and PTFE tubing	Norfloxacin	<1.5	95% confidence level	Medium - low	45	0.12 $mg\cdot L^{-1}$	0.45 $mg\cdot L^{-1}$	[33]
µPADs	40-350 $mg\cdot L^{-1}$	Sheet of filter paper	Hydroxylamine with 1,10-phenanthroline	10	95% confidence level	Affects copper	-	20 $mg\cdot L^{-1}$	40 $mg\cdot L^{-1}$	[35]
FIA	110-560 $mg\cdot L^{-1}$	-	fluorescence sensor (M141)	-	-	High	30	110 $mg\cdot L^{-1}$	-	[36]
FIA	0 – 0.5 $\mu g\cdot L^{-1}$	PTFE tubing	Oxidation reaction of N,N-dimethyl-p- phenylenediamine with hydrogen peroxide	<2	98-103	Medium	25	0.01 $\mu g\cdot L^{-1}$	0.07 $\mu g\cdot L^{-1}$	[45]
FIA	0.5-20 $mg\cdot L^{-1}$	PTFE	*Phyllanthus emblica*	<2.5	93.1 – 100.5	Only reacts with Fe(III)	90	0.31 $mg\cdot L^{-1}$	0.5 $mg\cdot L^{-1}$	[46]
SIA	0.1-2 $mg\cdot L^{-1}$	PVC pumping tube, and PTFE tubing	3,4-HPO ligands	<5	100±3	Medium	102	7 $\mu g\cdot L^{-1}$	24 $\mu g\cdot L^{-1}$	[47]
µFIA	25-6000 $mg\cdot L^{-1}$	PLA	Salicylic acid	3.1	99.9±1.1	High	6	11 $mg\cdot L^{-1}$	25 $mg\cdot L^{-1}$	This work
